# Lower insulin level is associated with sarcopenia in community-dwelling frail and non-frail older adults

**DOI:** 10.3389/fmed.2022.971622

**Published:** 2022-11-22

**Authors:** Yanxia Lu, Wee Shiong Lim, Xia Jin, Ma Schwe Zin Nyunt, Tamas Fulop, Qi Gao, Su Chi Lim, Anis Larbi, Tze Pin Ng

**Affiliations:** ^1^Department of Medical Psychology and Ethics, School of Basic Medical Sciences, Cheeloo College of Medicine, Shandong University, Jinan, China; ^2^Department of Geriatric Medicine, Tan Tock Seng Hospital, Singapore, Singapore; ^3^Lee Kong Chian School of Medicine, Nanyang Technological University, Singapore, Singapore; ^4^The Third Hospital of Jinan, Jinan, China; ^5^Gerontology Research Programme, Department of Psychological Medicine, National University Health System, Yong Loo Lin School of Medicine, National University of Singapore, Singapore, Singapore; ^6^Department of Medicine, Research Center on Aging, University of Sherbrooke, Sherbrooke, QC, Canada; ^7^Department of Endocrinology, Khoo Teck Puat Hospital, Singapore, Singapore; ^8^Biology of Ageing Laboratory, Singapore Immunology Network (SIgN), Agency for Science Technology and Research (A*STAR), Biopolis, Singapore, Singapore

**Keywords:** sarcopenia, diabetes, frailty, insulin, aging

## Abstract

**Background:**

Sarcopenia is common among older individuals with and without type 2 diabetes mellitus (T2DM). There are conflicting evidence in support of the role of insulin in the development of age-related and T2DM-related sarcopenia. We investigated the relationships between the levels of fasting insulin and other blood biomarkers related to insulin or lipid metabolism with the presence of sarcopenia in two independent studies.

**Materials and methods:**

In 246 pre-frail frail older individuals with (*n* = 41) and without T2DM (*n* = 205) in the Singapore Frailty Interventional Trial, sarcopenia was defined by low appendicular lean mass (ALM) relative to total body mass (skeletal muscle index, SMI = ALM/height^2^) and low lower limb strength or gait speed according to the Asian Working Group for Sarcopenia (AWGS) criteria released in 2019, and related to levels of fasting insulin and glucose, C-peptide, IGF-1, leptin, and active ghrelin. This investigation was validated in another independent study sample of 189 robust and pre-frail frail elderly in the Singapore Longitudinal Aging Study Wave 2 (SLAS-2).

**Results:**

Compared to non-sarcopenic individuals, those with sarcopenia and possible sarcopenia showed significantly lower fasting insulin (*p* < 0.05) in pre-frail/frail and non-frail older individuals. Consistent trends of relationships were observed for serum levels of C-peptide, IGF-1, leptin, and active ghrelin. In multivariable logistic regression models, sarcopenia was independently associated with low insulin (*p* < 0.05). Levels of fasting insulin, C-peptide, and leptin were also significantly associated with BMI, SMI, knee extension strength, gait speed, and physical activity score.

**Conclusion:**

Dysregulated insulin secretion in diabetic and non-diabetic older individuals may play an important role in age-related and diabetes-related sarcopenia.

## Introduction

Sarcopenia is an incipient manifestation of aging characterized by progressive loss of skeletal muscle mass and function. It is reported that sarcopenia predicts physical frailty, late-life disability, and mortality (1, 2). In a world with an accelerated population aging, the rapidly growing number of older individuals with sarcopenia and physical frailty makes it an important geriatric syndrome in clinical care. The prevalence of sarcopenia is 8–36% worldwide in individuals below 60 years and 10–27% in those equal to or above 60 years. Population-based prevalence of frailty varies by age, gender, and frailty classification, with 12% using physical frailty and 24% using the deficit accumulation model among those aged ≥50 years across 62 countries (3, 4). The underlying pathophysiology of sarcopenia is complex and far from being fully understood, involving nutritional deficiency, reduced physical activity, insulin resistance, atherosclerosis, and changes in inflammatory and endocrine functions (5, 6).

Disordered β cell functioning and insulin resistance are hallmarks of the development of type 2 diabetes mellitus (T2DM), which is prominently associated with sarcopenia (7). The association of insulin level and muscle mass and function decline in old age and those with T2DM is complex. Based on the classical view that insulin resistance is central to the development of T2DM, there are contrary hypotheses that insulin resistance facilitates the development of sarcopenia (8) and conversely that sarcopenia is a risk factor for insulin resistance and T2DM (9). Increasing evidence strongly points to disordered insulin secretion rather than insulin resistance playing a central role in driving the development of T2DM (10–12). Patients with diabetes may be vulnerable to sarcopenia due to primary changes in insulin level and activity (9, 13, 14). As an anabolic hormone, insulin stimulates protein synthesis *via* the uptake of amino acids into muscle tissues. Reduced insulin signaling in aging and diabetes hinders muscle protein synthesis (MPS) and promotes muscle protein degradation, leading to loss of muscle mass and eventually sarcopenia. Sarcopenia in turn further decreases the targeting mass of insulin action, reduces insulin sensitivity, and induces glucose dysregulation (15, 16).

Empirical evidence suggests the critical role of sufficient insulin in promoting protein synthesis, maintaining muscle function, and preventing muscle mass loss and sarcopenia (17, 18). Insulin exerts its anabolic action on skeletal muscles *via* stimulating glucose disposal (19) and inhibiting protein catabolism (20). A meta-analysis of human studies demonstrates that insulin has a permissive role in MPS in the presence of elevated amino acids and a major anti-catabolic effect in alleviating muscle protein breakdown (MPB) (21). In an interventional study, insulin treatmentattenuated annual decline of skeletal muscle index (SMI), especially in the lower extremities in Japanese patients with T2DM (22). There is an age-related decrease in insulin-mediated peripheral glucose utilization (23) and suppression of proteolysis (24) that are associated with sarcopenia. However, few studies have yet investigated the association of insulin and sarcopenia in older individuals with T2DM and those without (25, 26), with conflicting results generated. In the Baltimore Longitudinal Study of Aging, higher fasting and oral glucose tolerance test (OGTT) levels of insulin were associated with lower muscle mass in non-diabetic older individuals (27). On the other hand, Tanaka et al. (28), in a study of 191 male elderly with T2DM, suggested that endogenous insulin reduction is an independent risk factor of sarcopenia. Furthermore, supraphysiological hyperinsulinemia was reported to play an important role in the stimulation of MPS and anabolic signaling in the elderly (29). A possible explanation of the divergences may be related to the different ways of lean mass measurement (30). In individuals with sarcopenia, appendicular muscle mass index defined as muscle mass relative to height is mostly found positively associated with insulin resistance, while this correlation is negative in terms of muscle mass relative to body weight (31). Thus it is imperative to the standardized criteria to classify sarcopenia, in order to draw clear conclusion.

The present study aimed to ascertain whether sarcopenia is associated with the level of fasting insulin and other biomarkers related to insulin or lipid metabolism in diabetic and non-diabetic older individuals. Sarcopenia and physical frailty share core features such as impaired physical function, especially mobility, high prevalence, and intimate association with adverse health-related outcomes including disability and mortality in the elderly, and potential reversibility, and thus have been studied in parallel since the beginning (32, 33). We therefore studied the relationship of sarcopenia and fasting insulin level in a group of community-living pre-frail frail older persons. Furthermore, we validated and extended the results derived from this group to another independent study of elderly consisting of both robust and pre-frail frail elderly. We hypothesized that older individuals with sarcopenia may have lower levels of fasting insulin than those without sarcopenia, considering the anabolic role of insulin in maintaining muscle mass and function. The present study investigated for the first time, to the best of our knowledge, the association of sarcopenia and fasting insulin level in elderly with and without T2DM, and thus extend the findings from previous research through its broader relevance to community-living elderly without T2DM.

## Materials and methods

### Study design and participants

#### Participant recruitment

The subjects in the exploratory study were participants in the Singapore Frailty Interventional Trial, a randomized controlled trial of nutritional, physical, and cognitive interventions among community-living pre-frail frail older persons (clinicaltrial.gov identifier NCT00973258) (34). We used the baseline data from the participants who were 246 pre-frail or frail older individuals (aged 65 years and above) as determined from the Fried criteria for frailty syndrome (35) comprising of 5 components (unintentional weight loss, slowness, weakness, exhaustion, and physical inactivity). We validated the study in an independent validation sample of 189 robust and pre-frail frail elderly from Singapore Longitudinal Aging Study Wave 2 (SLAS-2) (36). The difference between the exploratory study and the validation study lies in that the exploratory study emphasizes 246 pre-frail or frail older individuals, and the validation study covers the full spectrum of 189 robust and pre-frail frail elderly. The participants from both studies were from the same Chinese Singaporean population.

#### Study design

In both studies, sarcopenia was determined from measures of muscle mass, strength, and function using dual-energy x-ray absorptiometry (DXA) whole body scan, Physiological Profile Assessment (PPA) (37), and the 6-meter fast gait speed test. Serum (in the exploratory study) or plasma (in the validation study) was isolated from blood samples and stored in −80°C until measurements. The studies were approved by National Health Group (NHG) Domain Specific Review Board (DSRB) of Singapore, and all participants provided written informed consent. All methods were performed in accordance with the approved protocol and relevant guidelines and regulations.

#### Settings

Community-living Singaporean Chinese.

#### Participant inclusion/exclusion criteria

**FIGURE 1:**
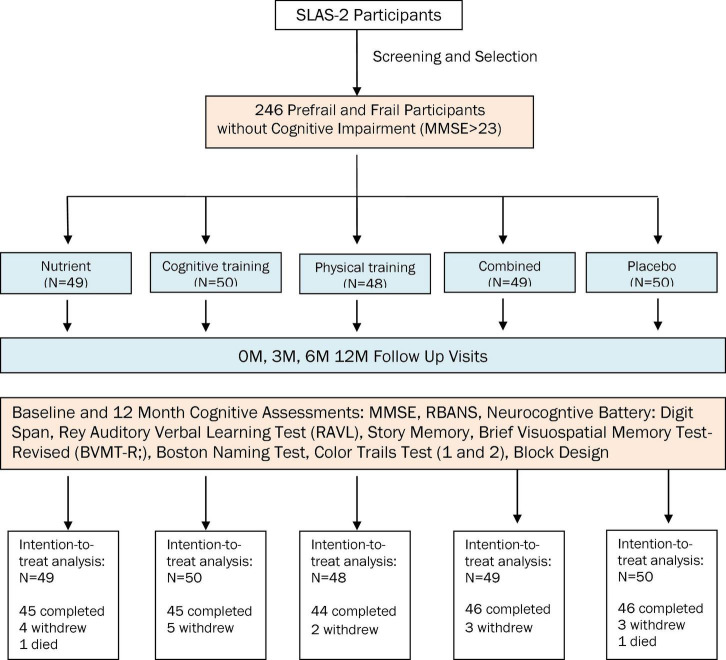
Participant recruitment flowchart in the exploratory study.

**FIGURE 2:**
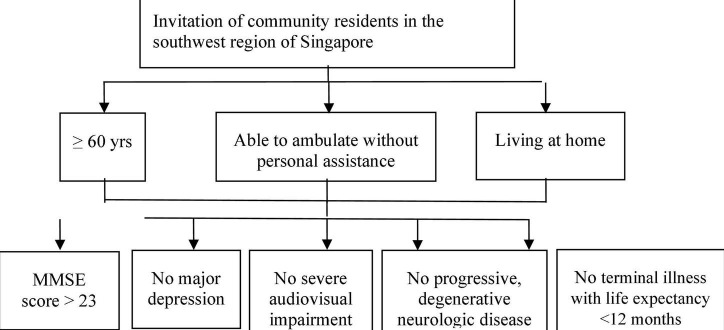
Participant recruitment flowchart in the validation study.

Inclusion criteria for both studies: (1) community residents in the southwest region of Singapore; (2) aged 65 years and above; (3) able to ambulate without personal assistance; (4) living at home. Exclusion criteria: (1) significant cognitive impairment (Mini Mental State Examination score ≤ 23); (2) major depression; (3) severe audiovisual impairment; (4) any progressive, degenerative neurologic disease; (5) terminal illness with life expectancy < 12 months. Additional inclusion criteria for the exploratory study: (1) prefrail and frail older adults identified based on Fried criteria defining physical frailty. Additional exclusion criteria for the exploratory study: (1) simultaneous participation in other interventional studies; (2) unavailable to participate for the full duration of the study. The recruitment procedures are summarized in Figures 1, 2, respectively, for the exploratory study and the validation study.

### Frailty measurements

Frailty was assessed by the presence of: (1) Involuntary or unintentional weight loss: body mass index (BMI) < 18.5 kg/m^2^ and/or unintentional weight loss > 10 pounds (4.5 kg) in the past 6 months; (2) Slowness: sex and height stratified lowest quintile values from the average of two measurements of the 6 m fast gait speed test; (3) Weakness: dominant knee extension strength measured from the average value from three trials using Lord’s strap and strain gauge assembly component of the PPA, falling within the lowest sex and BMI standardized quintile; (4) Exhaustion: exhaustion score was generated from the vitality domain in the SF-12 and those falling within the lowest quintile of energy score [below 10 out of 15, derived in a previous population-based study of frailty (38)] were classified as having exhaustion; (5) Physical inactivity: Physical activity was assessed by the Longitudinal Ageing Physical Activity Questionnaire (LAPAQ) which measured the frequency and duration (in minutes) of six different activities (walking outside, bicycling, gardening, light and heavy household activities, and sports activities) during the past 2 weeks. A participant was considered physically inactive if his/her overall average daily time spent on physical activities fell within the sex-specific lowest quintile. A participant with three and more components was determined to be frail, one or two components as pre-frail, and none of the components as robust.

### Dual-energy x-ray absorptiometry scans of body composition

Total and regional lean body mass and body fat was measured by dual energy X-ray absorptiometry (DXA) with the use of Hologic^®^ densitometer. Tests were performed by operators in accordance with the manufacturer’s protocol in Department of Diagnostic Radiology, National University Hospital of Singapore. The participant was instructed to lay in a supine position on the DXA table with limbs close to the body. The whole-body lean soft tissue mass was divided into the regions of arms, legs, and the trunk. Appendicular lean mass (ALM) was calculated by summing lean mass (kg) of the two upper limbs and two lower limbs, with the adjustment of limb cut lines according to specific anatomical landmarks as described by Heymsfield et al. (39).

### Measurement of lower limb strength

Lower limb strength was assessed by knee extension strength using the strap and strain gauge assembly component of the PPA described by Lord et al. (37), and a mean value from three trials (standardized by sex and BMI) was calculated. Low lower limb strength was classified as less than or equal to 18 kg for men and less than or equal to 16 kg for women.

### Gait speed test

The 6 m fast gait speed test was performed following standardized procedures described by Nelson et al. (40). Briefly, participants were instructed to stand with their toes touching the taped start line, and complete the 6 m walking as fast as possible. Total course time was recorded using a second watch from the moment their foot crossed the start line until the moment their foot crossed the stop line. The average speed of two trials was calculated and presented as meters/second (m/s).

### Definition of sarcopenia

Sarcopenia was identified in accordance with the recommendations from the Asian Working Group for Sarcopenia (AWGS) criteria released in 2019 (41). SMI was calculated as the ratio of ALM assessed by DXA scan over height squared. Participants were considered to have low SMI if the ratio was <7.0 kg/m^2^ in men or <5.4 kg/m^2^ in women. Low gait speed was defined as the average speed of two trials < 1.0 m/s. Participants were categorized as having sarcopenia if they had both low SMI and low lower limb strength or gait speed, possible sarcopenia if they had either low lower limb strength or gait speed but not low SMI, and non-sarcopenia if they had neither low SMI nor low lower limb strength or gait speed.

### Diabetes status

The presence of diabetes was ascertained by the subjects’ self-reports of a doctor’s diagnosis and treatment, with additional examination of their medication packages. Participants were determined as having diabetes when the use of appropriate diabetes medications was verified by the interviewer. The cross tables of number of participants with different status of sarcopenia and diabetes are summarized below:

Exploratory study:

**Table:** 

	No diabetes	Diabetes
Non-sarcopenia	25	3
Possible sarcopenia	98	21
Sarcopenia	82	17

Validation study:

**Table:** 

	No diabetes	Diabetes
Non-sarcopenia	28	4
Possible sarcopenia	49	15
Sarcopenia	79	14

### Blood biomarkers

Venous blood was drawn from overnight fasting participants and serum (in the exploratory study) or plasma (in the validation study) was isolated and stored in –80°C freezer until measurements. The levels of fasting insulin, C-peptide, leptin, and active ghrelin were tested using high throughput Luminex technology (Cat no.: HMHEMAG-34K-04 and HIGFMAG-52K-01; Millipore Corp., Billerica, MA, United States) following the manufacturers’ instructions. Experiments were performed by the platform operators. After an overnight incubation, the plates were read on a Flexmap 3D instrument (Luminex Corporation, Austin, TX, United States) and data were analyzed using Bioplex Manager 6.0 software (Bio-Rad Laboratories, Hercules, CA, United States). The concentration of IGF-1 was measured using Milliplex Map Human IGFI-II Magnetic Bead Panel (Cat no.: HIGFMAG-52K-01) in the exploratory sample and an enzyme-linked immunosorbent assay (ELISA) kit from Raybiotech Inc. (Norcross, GA, USA; Cat no.: ELH-IGF1) in the validation sample. The level of fasting glucose was measured by Department of Laboratory Medicine, National University Hospital of Singapore.

### Statistical analysis

Data analysis was performed using IBM SPSS 22 software (IBM, New York, NY, USA). We compared the differences in demographics, physical and functional status, and biomarkers among the sarcopenia, possible sarcopenia, and non-sarcopenia study participants using chi-squared test or one-way analysis of variance (ANOVA) with *post-hoc* Bonferroni correction for multiple comparisons as appropriate, in the whole group and among non-diabetic and diabetic participants separately. Logistic regressions were performed on each blood biomarker to estimate odds ratio of association with the presence of sarcopenia. Blood biomarkers were analyzed as continuous variables as well as in tertiles, with adjustment for age, sex, diabetes status, and percentage of whole body fat mass to control for potential confounding. *P* for trends across tertiles were calculated by assigning ordinal scores to the tertiles and repeating the logistic regressions. Linear regression models were used to examine the relations of each blood biomarker concentration to clinical and functional measures of sarcopenia and frailty (BMI, SMI, lower limb strength, exhaustion score, gait speed, and physical activity score) in the whole and stratified samples of non-diabetic and diabetic participants after adjusting for potential confounders. The level of statistical significance was set at *p* < 0.05 with a two-sided distribution.

## Results

The 246 pre-frail frail study participants in the exploratory study and the 189 robust and pre-frail frail elderly in the validation study were all Chinese and had average ages of 70.0 years (SD: 4.7 years) and 73.2 years (SD: 5.3 years), respectively. The exploratory and validatory samples had similar sex proportions, with 151 (61.4%) and 119 (63.0%) females, respectively. In the exploratory study, there were 41 (16.7%) elderly who had diabetes and 205 (83.3%) who did not have diabetes. The validatory study sample had a similar proportion of participants who had diabetes [33 (17.5%)]. Participants with diabetes had a mean disease history of 10 and 12 years, respectively, in the exploratory and validation studies, respectively, with all but five and four, respectively, taking anti-diabetic medications regularly. The commonest medication used was simvastatin and metformin, respectively. None of them were on insulin sensitizer drugs or treated with insulin.

In both studies, participants with sarcopenia had significantly lower BMI, SMI, knee extension strength, and gait speed in the whole sample and among subgroups of non-diabetic and diabetic individuals. Sarcopenia was associated with significantly higher exhaustion in the validation sample in the whole sample and among the subgroups (Table 1).

**TABLE 1:** Demographic, physical, and functional status of sarcopenia groups in all subjects.

		Sarcopenic subgroups			
		
		Sarcopenia	Possible sarcopenia	Non-sarcopenia	*P*
**Exploratory study**					
Sex (female)	151 (61.38)	60 (60.61)	82 (68.91)	9 (32.14)	0.002
Age (years)	70.03 ± 4.69	70.01 ± 4.66	70.08 ± 4.91	69.86 ± 3.97	0.973
Secondary and above education	75 (30.49)	34 (34.34)	30 (25.21)	11 (39.29)	0.194
BMI (kg/m^2^)	23.72 ± 3.48	21.47 ± 2.69**^+++^	25.60 ± 3.10**	23.72 ± 2.76	< 0.001
Skeletal muscle index (kg/m^2^)	6.11 ± 1.07	5.41 ± 0.86***^+++^	6.55 ± 0.87	6.79 ± 1.11	< 0.001
Knee extension strength (kg)	14.17 ± 4.98	13.02 ± 4.56***^+++^	13.05 ± 3.12***	22.92 ± 4.09	< 0.001
Gait speed (m/s)	0.95 ± 0.23	0.93 ± 0.23***	0.90 ± 0.21***	1.20 ± 1.82	< 0.001
Exhaustion score	10.63 ± 1.30	10.60 ± 1.38	10.63 ± 1.27	10.75 ± 1.14	0.858
Physical activity score	168.70 ± 112.20	162.40 ± 112.75	170.54 ± 103.50	183.19 ± 144.51	0.668
Fat mass (%, whole body)	32.88 ± 7.66	32.34 ± 8.01	34.39 ± 7.19***	28.59 ± 6.51	< 0.001
**Validation study**					
Sex (female)	119 (62.96)	59 (63.44)	49 (76.56)	11 (34.38)	< 0.001
Age (years)	73.16 ± 5.29	73.83 ± 5.32	72.31 ± 4.89	72.94 ± 5.84	0.204
Secondary and above education	50 (26.46)	24 (25.81)	12 (18.75)	14 (43.75)	0.032
BMI (kg/m^2^)	23.59 ± 3.61	21.35 ± 2.67***^+++^	26.14 ± 2.96	25.00 ± 3.12	< 0.001
Skeletal muscle index (kg/m^2^)	5.86 ± 1.04	5.26 ± 0.86***^+++^	6.39 ± 0.78	6.57 ± 0.97	< 0.001
Knee extension strength (kg)	13.89 ± 5.23	12.27 ± 3.99***	12.52 ± 4.37***	21.34 ± 3.28	< 0.001
Gait speed (m/s)	1.08 ± 0.30	1.07 ± 0.28***	0.98 ± 0.29***	1.32 ± 0.23	< 0.001
Exhaustion score	10.63 ± 2.09	10.40 ± 2.14***	10.27 ± 1.73***	12.06 ± 2.09	< 0.001
Physical activity score	243.84 ± 206.04	249.05 ± 188.05	209.26 ± 147.81	297.85 ± 320.23	0.131
Fat mass (%, whole body)	35.87 ± 6.33	34.90 ± 6.19^++^	38.19 ± 6.24**	34.04 ± 5.75	0.001

Data are presented as mean ± SD or number (percentage).

*** *P* < 0.001, ** *P* < 0.01 vs. the non-sarcopenia group; ^+++^ *P* < 0.001, ^++^ *P* < 0.01 vs. the possible sarcopenia group.

As shown in Table 2, sarcopenia was associated with lower fasting insulin (*p* < 0.05) in comparison to non-sarcopenia and possible sarcopenia in the whole exploratory sample of pre-frail/frail elderly. Among participants without diabetes, there was a positive trend of association between sarcopenia with lower fasting insulin, but this did not reach statistical significance, possibly due to the relatively large standard deviation (*p* = 0.055). In the validation study sample of robust and pre-frail/frail elderly, sarcopenic elderly had significantly lower insulin level than the non-sarcopenia and possible sarcopenia elderly in the whole sample and the non-diabetic group (*p* < 0.001). There was no difference in insulin levels among the sarcopenia subgroups of diabetic elderly, given the small sample size of the sarcopenic group. Lower C-peptide was observed in the sarcopenia group than in the non-sarcopenia and possible sarcopenia groups in the whole sample and the non-diabetic group. Sarcopenia was associated with lower leptin level than the non-sarcopenia group. No difference was observed in serum or plasma levels of fasting glucose, IGF-1 or active ghrelin among the sarcopenic subgroups (Supplementary Tables 1, 2).

**TABLE 2:** Blood biomarker concentrations of sarcopenia groups in frailty intervention trial (FIT) study subjects and Singapore longitudinal ageing studies (SLAS) subjects.

		Sarcopenic subgroups			
		
		Sarcopenia	Possible sarcopenia	Non-sarcopenia	*P*
**Exploratory study**					
Fasting insulin (pg/ml)	453.57 ± 318.30	375.90 ± 233.30*^+^	489.34 ± 328.09	565.32 ± 453.61	0.023
C-peptide (ng/ml)	1.54 ± 0.85	1.35 ± 0.63	1.64 ± 0.81	1.72 ± 1.39	0.067
Fasting glucose (mmol/L)	5.33 ± 1.28	5.35 ± 1.37	5.36 ± 1.29	5.16 ± 0.80	0.755
IGF-1 (ng/ml)	1.02 ± 0.74	1.02 ± 0.72	0.99 ± 0.76	1.12 ± 0.77	0.751
Leptin (ng/ml)	11.07 ± 10.05	8.92 ± 9.49^+^	13.16 ± 10.72	9.70 ± 7.27	0.029
Active ghrelin (pg/ml)	3.90 ± 4.51	3.47 ± 3.63	3.89 ± 4.72	5.38 ± 6.00	0.255
**Validation study**					
Fasting insulin (pg/ml)	307.92 ± 251.07	232.87 ± 206.93*^+^	395.47 ± 251.30	351.33 ± 303.28	< 0.001
C-peptide (ng/ml)	1.03 ± 0.45	0.90 ± 0.36*^+++^	1.17 ± 0.49	1.14 ± 0.53	< 0.001
Fasting glucose (mmol/L)	6.19 ± 1.57	6.23 ± 1.70	6.27 ± 1.53	5.94 ± 1.18	0.596
IGF-1 (ng/ml)	14.60 ± 7.15	15.36 ± 7.59	13.75 ± 6.97	14.47 ± 6.46	0.514
Leptin (ng/ml)	15.53 ± 16.23	10.96 ± 9.27^+++^	23.27 ± 21.87**	13.34 ± 13.39	< 0.001
Active ghrelin (pg/ml)	21.57 ± 16.96	22.39 ± 16.95	21.97 ± 16.70	18.42 ± 17.67	0.510

** *P* < 0.01, * *P* < 0.05 vs. the non-sarcopenia group; ^+++^ *P* < 0.001, ^+^ *P* < 0.05 vs. the possible sarcopenia group.

Table 3 shows the results of logistic regression analyses of the association of blood biomarkers with the presence of sarcopenia that were adjusted for age, sex, and diabetes status. The levels of insulin were borderlinely or significantly inversely associated with the presence of sarcopenia in the exploratory sample and the validation sample. The top tertile of fasting insulin was associated with lower risk of sarcopenia relative to the bottom tertile, with an OR of 0.297. A similar trend was observed in the validation study sample.

**TABLE 3:** Odds ratio of association of blood biomarkers with sarcopenia in whole sample.

	Per unit of Biomarker		Middle tertile vs. bottom tertile		Top tertile vs. bottom tertile		*P* (Trend)
				
	OR (95% CI)	*P*	OR (95% CI)	*P*	OR (95% CI)	*P*	
**Exploratory study**							
Insulin (pg/ml)	0.998 (0.996–1.000)	0.065	1.739 (0.381–7.947)	0.475	0.297 (0.080–1.111)	0.071	0.083
C-peptide (ng/ml)	0.724 (0.402–1.305)	0.283	1.367 (0.404–4.630)	0.615	1.359 (0.320–5.777)	0.678	0.641
Fasting glucose (mmol/L)	1.055 (0.589–1.892)	0.857	1.524 (0.483–4.805)	0.472	0.785 (0.221–2.792)	0.708	0.860
IGF-1 (ng/ml)	0.894 (0.421–1.899)	0.771	0.913 (0.235–3.543)	0.896	0.972 (0.252–3.745)	0.967	0.978
Leptin (ng/ml)	0.977 (0.915–1.043)	0.487	0.670 (0.156–2.878)	0.590	0.377 (0.069–2.044)	0.258	0.255
Active ghrelin (pg/ml)	0.927 (0.827–1.040)	0.199	1.017 (0.267–3.875)	0.980	0.819 (0.233–2.875)	0.756	0.749
**Validation study**							
Insulin (pg/ml)	0.998 (0.996–1.000)	0.047	0.750 (0.257–2.191)	0.599	0.372 (0.130–1.066)	0.066	0.069
C-peptide (ng/ml)	0.286 (0.099–0.827)	0.021	1.049 (0.340–3.239)	0.934	0.346 (0.118–1.018)	0.054	0.052
Fasting glucose (mmol/L)	1.232 (0.873–1.740)	0.236	1.155 (0.415–3.211)	0.783	1.401 (0.458–4.287)	0.554	0.553
IGF-1 (ng/ml)	1.008 (0.938–1.083)	0.829	0.814 (0.229–2.891)	0.750	0.836 (0.246–2.842)	0.774	0.768
Leptin (ng/ml)	0.952 (0.900–1.007)	0.086	0.572 (0.182–1.799)	0.339	0.291 (0.059–1.429)	0.128	0.131
Active ghrelin (pg/ml)	1.014 (0.984–1.045)	0.370	1.376 (0.514–3.683)	0.526	2.579 (0.815–8.159)	0.107	0.110

Data are adjusted for sex, age, status of diabetes, and percentage of whole body fat mass. *P* for trends across tertiles can be estimated by assigning ordinal scores of 1, 2, and 3 to the lowest, middle, and top tertile and repeating the logistic regressions. OR, odds ratio.

In linear regression models (Table 4), we found significant independent associations of blood biomarker concentrations with clinical and functional measures of sarcopenia and frailty. In the whole sample, the levels of fasting insulin and C-peptide were positively associated with BMI, SMI, and knee extension strength. IGF-1 was positively associated with knee extension strength. We also found that the level of leptin was positively associated with BMI, SMI, and physical activity score. Similar trends were observed in both non-diabetic and diabetic subgroups as shown in Supplementary Tables 3, 4, respectively.

**TABLE 4:** Relations of blood biomarker concentrations to clinical and functional measures of sarcopenia and frailty.

	BMI (kg/m^2^)			Skeletal muscle index (kg/m^2^)		
		
	b ± SE	β	*P*	b ± SE	β	*P*
**Exploratory study**						
Fasting insulin (pg/ml)	0.004 ± 0.001	0.318	< 0.001	0.572 ± 0.195	0.167	0.004
C-peptide (ng/ml)	1.456 ± 0.315	0.339	< 0.001	203.419 ± 73.133	0.159	0.006
Fasting glucose (mmol/L)	0.421 ± 0.232	0.152	0.071	56.963 ± 54.743	0.067	0.299
IGF-1 (ng/ml)	0.619 ± 0.374	0.127	0.099	117.567 ± 84.276	0.081	0.165
Leptin (ng/ml)	0.183 ± 0.027	0.510	< 0.001	22.689 ± 6.567	0.210	0.001
Active ghrelin (pg/ml)	0.010 ± 0.063	0.012	0.875	13.174 ± 14.102	0.055	0.352
**Validation study**						
Fasting insulin (pg/ml)	0.005 ± 0.001	0.325	< 0.001	0.001 ± 0.000	0.239	< 0.001
C-peptide (ng/ml)	3.337 ± 0.543	0.418	< 0.001	0.562 ± 0.126	0.245	< 0.001
Fasting glucose (mmol/L)	0.281 ± 0.187	0.122	0.135	0.024 ± 0.042	0.036	0.566
IGF-1 (ng/ml)	−0.043 ± 0.045	–0.086	0.337	−0.008 ± 0.010	–0.060	0.407
Leptin (ng/ml)	0.127 ± 0.015	0.570	< 0.001	0.015 ± 0.004	0.229	< 0.001
Active ghrelin (pg/ml)	−0.020 ± 0.016	–0.094	0.210	−0.005 ± 0.004	–0.076	0.195

	**Knee extension strength (kg)**			**Exhaustion score**		

**Exploratory study**						
Fasting insulin (pg/ml)	0.002 ± 0.001	0.142	0.038	0.000 ± 0.000	–0.026	0.742
C-peptide (ng/ml)	1.129 ± 0.422	0.180	0.008	0.042 ± 0.115	0.029	0.715
Fasting glucose (mmol/L)	0.091 ± 0.293	0.023	0.756	0.035 ± 0.083	0.036	0.671
IGF-1 (ng/ml)	1.240 ± 0.477	0.174	0.010	0.008 ± 0.132	0.005	0.953
Leptin (ng/ml)	0.033 ± 0.039	0.063	0.399	0.013 ± 0.010	0.102	0.224
Active ghrelin (pg/ml)	0.052 ± 0.081	0.044	0.521	−0.014 ± 0.022	–0.051	0.517
**Validation study**						
Fasting insulin (pg/ml)	−0.001 ± 0.001	–0.043	0.534	−0.001 ± 0.001	–0.077	0.291
C-peptide (ng/ml)	0.905 ± 0.799	0.078	0.259	−0.390 ± 0.340	–0.084	0.253
Fasting glucose (mmol/L)	0.042 ± 0.254	0.012	0.870	0.215 ± 0.107	0.161	0.045
IGF-1 (ng/ml)	0.050 ± 0.057	0.072	0.380	−0.005 ± 0.026	–0.016	0.857
Leptin (ng/ml)	0.021 ± 0.023	0.064	0.378	0.003 ± 0.010	0.026	0.736
Active ghrelin (pg/ml)	−0.006 ± 0.022	–0.018	0.795	0.005 ± 0.009	0.044	0.559

	**Gait speed (cm/s)**			**Physical activity score**		

**Exploratory study**						
Fasting insulin (pg/ml)	−0.009 ± 0.006	0.125	0.103	−0.048 ± 0.026	–0.143	0.066
C-peptide (ng/ml)	1.831 ± 2.153	0.065	0.396	−15.974 ± 9.735	–0.126	0.103
Fasting glucose (mmol/L)	0.296 ± 1.512	0.016	0.845	−7.635 ± 7.226	–0.088	0.292
IGF-1 (ng/ml)	−1.356 ± 2.441	–0.043	0.579	3.498 ± 11.073	0.024	0.752
Leptin (ng/ml)	−0.107 ± 0.197	–0.045	0.589	−1.944 ± 0.882	–0.182	0.029
Active ghrelin (pg/ml)	−0.511 ± 0.404	–0.097	0.208	1.888 ± 1.841	0.080	0.307
**Validation study**						
Fasting insulin (pg/ml)	0.003 ± 0.009	0.026	0.726	0.044 ± 0.061	0.053	0.475
C-peptide (ng/ml)	−3.945 ± 4.811	–0.060	0.413	17.962 ± 33.860	0.039	0.596
Fasting glucose (mmol/L)	−1.188 ± 1.522	–0.063	0.436	2.904 ± 10.715	0.022	0.787
IGF-1 (ng/ml)	−0.214 ± 0.373	–0.050	0.567	2.036 ± 2.405	0.074	0.399
Leptin (ng/ml)	−0.144 ± 0.141	–0.079	0.307	−0.856 ± 0.992	–0.067	0.390
Active ghrelin (pg/ml)	0.026 ± 0.125	0.015	0.837	0.398 ± 0.917	0.033	0.665

Data are adjusted for sex, age, and status of diabetes.

## Discussion

This study shows for the first time that the association of sarcopenia with low fasting insulin in the elderly is independent of whether the subjects have diabetes or not. Furthermore, fasting insulin, C-peptide, and leptin were significantly associated with one or more clinical and functional measures of sarcopenia and frailty including total body mass, muscle mass, knee extension strength, gait speed, and physical activity. The results are consistent with studies which showed that measures of endogenous insulin secretion were inversely associated with the presence of sarcopenia in individuals with type 2 diabetes (28) and that insulin treatment attenuates skeletal muscle mass loss (21, 22). Our results suggest that insulin is clearly involved in the biological process of sarcopenia (18, 42) in the elderly.

The mechanisms underlying low insulin in sarcopenia in diabetic and non-diabetic older individuals is not completely understood. The hypothesis that age-related changes in the number and size of Type I versus Type 2 muscle fibers favoring a tendency for altered insulin sensitivity may explain the observed association in this study (9). In diabetes, the pathogenetic role and mechanisms of insulin is highly complex, and a large volume of literature now strongly favor the view that β cell dysfunction but not insulin resistance may be the central defect responsible for the development of Type 2 diabetes (11). Disordered insulin secretion contributes to the development of insulin resistance and may be an initiating factor in the progression to T2DM (43, 44). The loss of β cell function is progressive throughout the course of diabetes, beginning with defect in first-phase insulin secretion, followed by a decreasing maximal capacity of insulin secretion, before a reduction of steady-state and basal insulin secretion and complete β cell failure requiring insulin treatment (45). Reduction of insulin level and dysregulated insulin signaling activity both in diabetic or non-diabetic older individuals diminishes the anabolic capacity of insulin to alleviate MPB in skeletal muscles and may possibly be a primary mechanism shared by age-related sarcopenia and diabetes.

Taken together, a reduced level of endogenous insulin secretion may thus be a risk factor for sarcopenia development in both Japanese and Chinese diabetic elderly. This is particularly so given that it is widely recognized that T2DM in East Asians is characterized primarily by β cell dysfunction (12). As diabetes is a highly heterogeneous condition across different populations, whether this may explain the contrary findings from Western population studies of non-diabetic older persons (27, 46), showing hyperinsulinemia to be associated with lower muscle mass and strength, should be investigated in further studies.

IGF-1 has been shown to have an anabolic effect on muscle tissues. In line with report from previous studies (28, 47), our study found that the level of IGF-1 was associated with increased muscle strength. The serum level of IGF-1 decreases during aging, together with decreased tissue responsiveness and intracellular signaling (48). Previous studies also reported IGF-1 to be associated with various physical performance and mobility tasks, and all-cause mortality in the elderly (49, 50). In mouse models, exogenous systemic administration of IGF-1 improves contractile function, facilitates the functional recovery of injured skeletal muscle, and enhances muscle oxidative enzymes (51, 52). This may be explained by the mediating role of IGF-1 in muscle growth and subsequent regeneration: IGF-1 promotes the stimulation of muscle cell proliferation and differentiation, and MPS while inhibiting its degradation (53). The levels of c-peptide are found lower in participants with sarcopenia and positively associated with BMI, SMI, and knee extension strength in the whole sample and those without diabetes. C-peptide level is not affected by insulin injections or liver metabolization and are thus considered a better measure of portal insulin secretion than insulin itself (54, 55). Leptin is an adipokine secreted by white adipose tissue. Leptin acts on hypothalamic neurons to regulate food consumption and body fat (56). Moreover, leptin impacts on pancreatic beta cells, immune cells and muscle cells and modulate glucose metabolism, inflammatory processes and insulin resistance (57, 58). Considering the proinflammatory properties of leptin, previous studies have investigated the relationship between serum/plasma leptin level and sarcopenia, with generation of conflicting results. Our results of lower leptin level in sarcopenic older individuals corroborate the findings of previous studies which demonstrated an inverse association between serum leptin level and sarcopenia in hemodialysis patients (59). Consistently, frail hospitalized patients with a lower mid-arm muscle area were found to have lower leptin levels than their healthy counterparts (60). The discrepancies in these studies may be caused by disparate effects of leptin on different populations such as obese individuals, hemodialysis subjects, and individuals with different ethnicities. These findings taken together thus also support the direct and indirect role of insulin in increasing muscle mass and function, regardless of diabetes status.

The present study defined sarcopenia according to the latest population-specific standardized criteria for Asian elderly: AWGS 2019. Furthermore, we validated our results derived from pre-frail frail older individuals in another independent study sample of robust and pre-frail frail older individuals, thus supporting the robustness of these findings. However, there are limitations in the cross-sectional study design and future studies employing longitudinal measurements should be undertaken to elucidate its temporal causal relationship. There is also limited sample size in this study. Insulin secretion and impaired insulin signaling may be a primary defect in sarcopenia associated with their prognosis and quality of life in both diabetic and non-diabetic individuals. Moderate-intensity exercise-based interventions, in combination with appropriate nutritional supplementation, are recommended to improve both muscle mass and performance and insulin levels in the elderly.

## Data availability statement

The raw data supporting the conclusions of this article will be made available by the authors, without undue reservation.

## Ethics statement

The studies involving human participants were reviewed and approved by National Health Group (NHG) Domain Specific Review Board (DSRB) of Singapore. The patients/participants provided their written informed consent to participate in this study.

## Author contributions

YL did the statistical analyses and drafted the manuscript. TN designed the study and directed the data collection as the principal investigator of the study. All authors contributed to the study concept and design, interpretation of data, and critical review of the manuscript and approved the final version.
