# Supplementary material for: Lower insulin level is associated with sarcopenia in community-dwelling frail and non-frail older adults
Source: Front Med (Lausanne). 2022 Nov 22;9:971622. doi: 10.3389/fmed.2022.971622 (PMC9722960; doi:10.3389/fmed.2022.971622)
Supplement: Supplementary file 1 [file Table_1.pdf]

Supplementary Material

Table S1. Demographic, physical and functional status of sarcopenia groups in diabetic and non-diabetic elderly

		Sarcopenic subgroups			
		**Sarcopenia**	**Possible sarcopenia**	**Non-sarcopenia**	**P**
Exploratory study					
Non-Diabetic					
Sex (female)	123 (60.00)	50 (60.98)	65 (66.33)	8 (32.00)	0.007
Age (years)	70.04 ± 4.77	70.30 ± 4.93	69.94 ± 4.89	69.56 ± 3.77	0.761
Secondary and above education	66 (32.20)	29 (35.37)	27 (27.55)	10 (40.00)	0.360
BMI (kg/m^2)	23.72 ± 3.48	21.32 ± 2.88^{**+++}	25.72 ± 3.23*	23.71 ± 2.82	<0.001
Skeletal muscle index (kg/m^2)	6.11 ± 1.07	5.37 ± 0.89^{***+++}	6.59 ± 0.90	6.80 ± 1.09	<0.001
Knee extension strength (kg)	14.17 ± 4.98	12.98 ± 4.82***	13.07 ± 3.16***	22.94 ± 4.29	<0.001
Gait speed (m/s)	0.95 ± 0.23	0.92 ± 0.23***	0.92 ± 0.21***	1.18 ± 0.14	<0.001
Exhaustion score	10.63 ± 1.30	10.57 ± 1.46	10.66 ± 1.20	10.72 ± 1.21	0.847
Physical activity score	168.70 ± 112.20	228.75 ± 128.29	212.40 ± 142.87	185.77 ± 167.60	0.414
Fat mass (%, whole body)	32.94 ± 8.06	32.45 ± 8.40	34.56 ± 7.62**	28.47 ± 6.78	0.002
Diabetic					
Sex (female)	28 (68.29)	10 (58.82)	17 (80.95)	1 (33.33)	0.139
Age (years)	69.98 ± 4.36	68.59 ± 2.69	70.76 ± 5.09	72.33 ± 5.69	0.197
Secondary and above education	9 (21.95)	5 (29.41)	3 (14.29)	1 (33.33)	0.473
BMI (kg/m^2)	23.78 ± 2.43	22.18 ± 1.33^{+++}	25.07 ± 2.40	23.80 ± 2.69	<0.001
Skeletal muscle index (kg/m^2)	6.07 ± 0.83	5.62 ± 0.70^{+}	6.35 ± 0.67	6.71 ± 1.53	0.007
Knee extension strength (kg)	13.79 ± 3.92	13.20 ± 3.16***	12.98 ± 3.01***	22.78 ± 2.22	<0.001
Gait speed (m/s)	0.91 ± 0.26	0.97 ± 0.24*	0.80 ± 0.18**	1.32 ± 0.42	0.001
Exhaustion score	10.61 ± 1.26	10.71 ± 0.92	10.48 ± 1.57	11.00 ± 0.00	0.743
Physical activity score	177.95 ± 115.52	178.50 ± 98.60	246.48 ± 136.95	305.48 ± 226.25	0.166
Fat mass (%, whole body)	32.59 ± 5.33	31.80 ± 5.92	33.65 ± 4.88	29.58 ± 4.39	0.347
Validation study					
Non-Diabetic					
Sex (female)	97 (62.18)	51 (64.56)	36 (73.47)	10 (35.71)	0.004
Age (years)	72.86 ± 5.20	73.51 ± 5.41	71.73 ± 4.57	73.00 ± 5.48	0.171
Secondary and above education	46 (29.49)	22 (27.85)	10 (20.41)	14 (50.00)	0.021
BMI (kg/m^2)	23.51 ± 3.69	21.32 ± 2.77^{***+++}	26.23 ± 3.23	24.94 ± 2.81	<0.001
Skeletal muscle index (kg/m^2)	5.85 ± 1.05	5.23 ± 0.86^{***+++}	6.43 ± 0.78	6.57 ± 0.91	<0.001
Knee extension strength (kg)	13.97 ± 5.37	12.31 ± 4.20***	12.50 ± 4.57***	21.26 ± 3.16	<0.001
Gait speed (m/s)	1.09 ± 0.30	1.06 ± 0.27***	1.00 ± 0.30***	1.34 ± 0.24	<0.001
Exhaustion score	10.68 ± 2.13	10.49 ± 2.17**	10.22 ± 1.71**	12.00 ± 2.21	<0.001
Physical activity score	251.27 ± 213.76	254.91 ± 196.58	224.07 ± 150.27	288.59 ± 328.18	0.437
Fat mass (%, whole body)	35.94 ± 6.41	35.20 ± 6.14^{+}	38.18 ± 6.61*	34.09 ± 5.91	0.009
Diabetic					
Sex (female)	22 (66.67)	8 (57.14)	13 (86.67)	1 (25.00)	0.041
Age (years)	74.61 ± 5.54	75.64 ± 4.52	74.20 ± 5.58	72.50 ± 9.04	0.578
Secondary and above education	4 (12.12)	2 (14.29)	2 (13.33)	0 (0.00)	0.728
BMI (kg/m^2)	23.96 ± 3.24	21.53 ± 2.07^{*+++}	25.85 ± 1.88	25.43 ± 5.38	<0.001
Skeletal muscle index (kg/m^2)	5.94 ± 1.00	5.45 ± 0.86	6.23 ± 0.80	6.57 ± 1.56	0.038
Knee extension strength (kg)	13.49 ± 4.57	12.07 ± 2.52***	12.58 ± 3.77***	21.92 ± 4.54	<0.001
Gait speed (m/s)	1.06 ± 0.29	1.17 ± 0.32	0.92 ± 0.22	1.24 ± 1.71	0.027
Exhaustion score	10.42 ± 1.95	9.86 ± 1.96	10.40 ± 1.84	12.50 ± 1.00	0.053
Physical activity score	208.74 ± 162.94	216.02 ± 130.41	160.90 ± 132.76	362.68 ± 289.77	0.083
Fat mass (%, whole body)	35.54 ± 6.06	33.18 ± 6.41	38.23 ± 5.06	33.70 ± 5.30	0.061

Data are presented as mean ± SD or number (percentage). *** P < 0.001, ** P < 0.01, * P < 0.05 vs. the non-sarcopenia group; $^{+++}$ P < 0.001, $^{+}$ P < 0.05 vs. the possible sarcopenia group.

Table S2. Blood biomarker concentrations of sarcopenia groups in in diabetic and non-diabetic elderly

		Sarcopenic subgroups			
		**Sarcopenia**	**Possible sarcopenia**	**Non-sarcopenia**	**P**
Exploratory study					
Non-Diabetic					
Fasting insulin (pg/ml)	439.34 ± 310.96	363.35 ± 233.52	473.83 ± 303.68	536.67 ± 482.90	0.055
C-peptide (ng/ml)	1.54 ± 0.85	1.34 ± 0.66	1.61 ± 0.82	1.67 ± 1.48	0.172
Fasting glucose (mmol/L)	5.33 ± 1.28	4.87 ± 0.56	5.12 ± 1.14	4.96 ± 0.54	0.192
IGF-1 (ng/ml)	1.02 ± 0.74	0.95 ± 0.69	1.01 ± 0.71	1.12 ± 0.66	0.691
Leptin (ng/ml)	11.07 ± 10.05	9.52 ± 10.31	12.93 ± 10.48	9.67 ± 7.80	0.146
Active ghrelin (pg/ml)	3.90 ± 4.51	3.61 ± 3.83	3.91 ± 4.31	5.37 ± 6.22	0.357
Diabetic					
Fasting insulin (pg/ml)	525.75 ± 350.29	428.04 ± 234.19	581.10 ± 455.91	727.70 ± 206.43	0.327
C-peptide (ng/ml)	1.64 ± 0.66	1.40 ± 0.49	1.81 ± 0.74	2.01 ± 0.87	0.184
Fasting glucose (mmol/L)	7.03 ± 1.57	7.51 ± 1.82	0.65 ± 1.34	6.70 ± 0.82	0.277
IGF-1 (ng/ml)	1.11 ± 0.96	1.33 ± 0.79	0.86 ± 1.03	1.16 ± 1.48	0.500
Leptin (ng/ml)	10.25 ± 9.33	6.40 ± 4.15	14.51 ± 12.45	9.90 ± 3.80	0.091
Active ghrelin (pg/ml)	3.55 ± 5.07	2.90 ± 2.65	3.77 ± 6.92	5.45 ± 5.75	0.735
Validation study					
Non-Diabetic					
Fasting insulin (pg/ml)	298.32 ± 252.41	215.68 ± 206.46^{**+++}	385.34 ± 243.16	376.25 ± 311.34	<0.001
C-peptide (ng/ml)	1.00 ± 0.43	0.88 ± 0.35^{**++}	1.10 ± 0.42	11.49 ± 0.55	0.002
Fasting glucose (mmol/L)	5.89 ± 1.27	5.95 ± 1.49	5.91 ± 1.10	5.70 ± 0.81	0.669
IGF-1 (ng/ml)	14.35 ± 7.18	14.82 ± 7.50	13.69 ± 7.32	14.44 ± 6.46	0.775
Leptin (ng/ml)	14.90 ± 13.90	11.01 ± 9.16^{+++}	21.71 ± 17.36*	13.98 ± 14.20	<0.001
Active ghrelin (pg/ml)	22.68 ± 18.22	23.38 ± 17.98	23.60 ± 18.37	19.09 ± 18.83	0.517
Diabetic					
Fasting insulin (pg/ml)	354.42 ± 242.95	328.64 ± 188.67	430.92 ± 284.84	176.87 ± 177.99	0.160
C-peptide (ng/ml)	1.19 ± 0.53	1.01 ± 0.39	1.38 ± 0.63	1.09 ± 0.42	0.169
Fasting glucose (mmol/L)	7.62 ± 1.99	7.81 ± 1.98	7.45 ± 2.11	7.63 ± 2.05	0.891
IGF-1 (ng/ml)	15.74 ± 6.89	17.66 ± 7.88	13.95 ± 5.98	14.78 ± 9.16	0.475
Leptin (ng/ml)	18.50 ± 24.50	10.69 ± 10.22	28.36 ± 32.90	8.89 ± 2.82	0.105
Active ghrelin (pg/ml)	16.20 ± 6.32	16.42 ± 5.76	16.66 ± 7.56	13.75 ± 1.98	0.720

** $P < 0.01$, * $P < 0.05$ vs. the non-sarcopenia group; $^{+++}$ $P < 0.001$ vs. the possible sarcopenia group.

Table S3. Relations of blood biomarker concentrations to clinical and functional measures of sarcopenia and frailty among non-diabetic subjects

	BMI (kg/m²)			Skeletal muscle index (kg/m²)		
	b ± SE	β	P	b ± SE	β	P
Exploratory study (N=205)						
Fasting insulin (pg/ml)	0.005 ± 0.001	0.369	<0.001	0.610 ± 0.223	0.169	0.007
C-peptide (ng/ml)	1.540 ± 0.345	0.355	<0.001	188.095 ± 79.284	0.148	0.019
Fasting glucose (mmol/L)	0.890 ± 0.306	0.214	0.004	147.240 ± 71.599	0.116	0.041
IGF-1 (ng/ml)	1.230 ± 0.450	0.227	0.007	243.026 ± 99.774	0.153	0.016
Leptin (ng/ml)	0.200 ± 0.030	0.542	<0.001	23.209 ± 7.310	0.212	0.002
Active ghrelin (pg/ml)	0.032 ± 0.074	0.037	0.670	7.264 ± 16.331	0.029	0.657
Validation study (N=156)						
Fasting insulin (pg/ml)	0.005 ± 0.001	0.355	<0.001	0.001 ± 0.000	0.269	<0.001
C-peptide (ng/ml)	3.552 ± 0.643	0.413	<0.001	0.579 ± 0.146	0.237	<0.001
Fasting glucose (mmol/L)	0.391 ± 0.236	0.135	0.101	0.048 ± 0.052	0.058	0.354
IGF-1 (ng/ml)	-0.047 ± 0.051	-0.091	0.359	-0.007 ± 0.011	-0.047	0.555
Leptin (ng/ml)	0.180 ± 0.018	0.679	<0.001	0.023 ± 0.005	0.303	<0.001
Active ghrelin (pg/ml)	-0.018 ± 0.017	-0.087	0.290	-0.005 ± 0.004	-0.082	0.193

	Knee extension strength (kg)			Exhaustion score		
	b ± SE	β	P	b ± SE	β	P
Exploratory study (N=205)						
Fasting insulin (pg/ml)	0.003 ± 0.001	0.152	0.039	0.000 ± 0.000	-0.045	0.597
C-peptide (ng/ml)	1.170 ± 0.453	0.187	0.011	0.017 ± 0.123	0.011	0.893
Fasting glucose (mmol/L)	-0.075 ± 0.382	-0.013	0.845	0.083 ± 0.104	0.060	0.427
IGF-1 (ng/ml)	1.051 ± 0.574	0.134	0.069	0.103 ± 0.156	0.056	0.509
Leptin (ng/ml)	0.051 ± 0.043	0.095	0.237	0.011 ± 0.011	0.091	0.325
Active ghrelin (pg/ml)	0.029 ± 0.093	0.023	0.757	0.001 ± 0.025	0.003	0.971
Validation study (N=156)						
Fasting insulin (pg/ml)	0.000 ± 0.002	0.018	0.818	-0.001 ± 0.001	-0.104	0.196
C-peptide (ng/ml)	1.334 ± 0.949	0.107	0.162	-0.457 ± 0.397	-0.092	0.252
Fasting glucose (mmol/L)	0.164 ± 0.323	0.039	0.612	0.115 ± 0.135	0.069	0.394
IGF-1 (ng/ml)	0.091 ± 0.065	0.125	0.164	0.008 ± 0.029	0.027	0.778
Leptin (ng/ml)	0.031 ± 0.032	0.080	0.328	0.007 ± 0.013	0.048	0.578
Active ghrelin (pg/ml)	-0.005 ± 0.023	-0.015	0.841	0.008 ± 0.009	0.066	0.419

	Gait speed (cm/s)			Physical activity score		
	b ± SE	β	P	b ± SE	β	P
Exploratory study (N=205)						
Fasting insulin (pg/ml)	0.008 ± 0.006	0.104	0.216	-0.049 ± 0.029	-0.142	0.093
C-peptide (ng/ml)	2.437 ± 2.166	0.094	0.262	-14.739 ± 10.283	-0.120	0.154
Fasting glucose (mmol/L)	-2.653 ± 1.846	-0.105	0.152	-18.938 ± 9.109	-0.154	0.039
IGF-1 (ng/ml)	-3.217 ± 2.733	-0.099	0.241	4.907 ± 12.981	0.032	0.706
Leptin (ng/ml)	-0.075 ± 0.204	-0.034	0.714	-2.006 ± 0.959	-0.190	0.038
Active ghrelin (pg/ml)	-0.385 ± 0.436	-0.074	0.380	1.263 ± 2.081	0.052	0.545
Validation study (N=156)						
Fasting insulin (pg/ml)	0.008 ± 0.009	0.068	0.394	0.051 ± 0.069	0.060	0.459
C-peptide (ng/ml)	-0.883 ± 5.593	-0.013	0.875	34.487 ± 40.306	0.069	0.394
Fasting glucose (mmol/L)	-3.695 ± 1.870	-0.158	0.050	-5.822 ± 13.669	-0.035	0.671
IGF-1 (ng/ml)	-0.177 ± 0.412	-0.041	0.669	1.025 ± 2.664	0.037	0.701
Leptin (ng/ml)	-0.075 ± 0.185	-0.035	0.684	-0.384 ± 1.337	-0.025	0.774
Active ghrelin (pg/ml)	0.026 ± 0.132	0.016	0.847	0.456 ± 0.956	0.039	0.634

Data are adjusted for sex and age.

Table S4. Relations of blood biomarker concentrations to clinical and functional measures of sarcopenia and frailty among diabetic subjects

	BMI (kg/m^2)			Skeletal muscle index (kg/m^2)		
	b ± SE	β	P	b ± SE	β	P
Exploratory study (N=41)						
Fasting insulin (pg/ml)	0.000 ± 0.002	0.029	0.888	0.412 ± 0.398	0.156	0.312
C-peptide (ng/ml)	0.716 ± 0.792	0.180	0.375	354.717 ± 199.047	0.255	0.087
Fasting glucose (mmol/L)	-0.393 ± 0.272	-0.248	0.158	-129.403 ± 73.387	-0.237	0.087
IGF-1 (ng/ml)	-1.002 ± 0.524	-0.363	0.068	-256.905 ± 138.326	-0.267	0.076
Leptin (ng/ml)	0.087 ± 0.059	0.306	0.153	21.812 ± 15.553	0.220	0.174
Active ghrelin (pg/ml)	-0.066 ± 0.106	-0.125	0.541	30.444 ± 27.285	0.167	0.276
Validation study (N=33)						
Fasting insulin (pg/ml)	0.003 ± 0.002	0.193	0.294	0.000 ± 0.001	0.101	0.506
C-peptide (ng/ml)	2.774 ± 0.980	0.453	0.008	0.476 ± 0.266	0.252	0.084
Fasting glucose (mmol/L)	0.123 ± 0.297	0.076	0.682	-0.019 ± 0.076	-0.038	0.802
IGF-1 (ng/ml)	-0.013 ± 0.091	-0.034	0.885	-0.008 ± 0.024	-0.069	0.739
Leptin (ng/ml)	0.060 ± 0.023	0.452	0.014	0.004 ± 0.006	0.109	0.492
Active ghrelin (pg/ml)	-0.107 ± 0.093	-0.210	0.261	0.009 ± 0.024	0.056	0.720

	Knee extension strength (kg)			Exhaustion score		
	b ± SE	β	P	b ± SE	β	P
Exploratory study (N=41)						
Fasting insulin (pg/ml)	0.001 ± 0.002	0.086	0.636	0.000 ± 0.001	0.071	0.731
C-peptide (ng/ml)	0.943 ± 1.157	0.143	0.423	0.248 ± 0.353	0.141	0.489
Fasting glucose (mmol/L)	0.107 ± 0.424	0.042	0.803	-0.025 ± 0.152	-0.030	0.870
IGF-1 (ng/ml)	1.528 ± 0.757	0.335	0.055	-0.214 ± 0.245	-0.175	0.392
Leptin (ng/ml)	-0.084 ± 0.088	-0.179	0.350	0.024 ± 0.027	0.193	0.374
Active ghrelin (pg/ml)	0.081 ± 0.154	0.094	0.605	-0.065 ± 0.045	-0.280	0.164
Validation study (N=33)						
Fasting insulin (pg/ml)	-0.008 ± 0.003	-0.394	0.015	0.001 ± 0.001	0.086	0.642
C-peptide (ng/ml)	-0.415 ± 1.439	-0.048	0.775	-0.354 ± 0.663	-0.096	0.597
Fasting glucose (mmol/L)	-0.170 ± 0.387	-0.074	0.664	0.431 ± 0.161	0.439	0.012
IGF-1 (ng/ml)	-0.149 ± 0.118	-0.275	0.219	-0.059 ± 0.061	-0.223	0.339
Leptin (ng/ml)	0.010 ± 0.033	0.056	0.756	0.000 ± 0.015	0.004	0.985
Active ghrelin (pg/ml)	-0.043 ± 0.125	-0.060	0.732	-0.067 ± 0.057	-0.215	0.249

	Gait speed (cm/s)			Physical activity score		
	b ± SE	β	P	b ± SE	β	P
Exploratory study (N=41)						
Fasting insulin (pg/ml)	0.014 ± 0.016	0.180	0.380	-0.060 ± 0.056	-0.196	0.299
C-peptide (ng/ml)	-3.520 ± 8.490	-0.083	0.682	-24.534 ± 29.449	-0.153	0.413
Fasting glucose (mmol/L)	5.239 ± 2.998	0.302	0.090	8.091 ± 12.315	0.110	0.516
IGF-1 (ng/ml)	2.639 ± 5.927	0.090	0.660	-10.053 ± 20.762	-0.091	0.633
Leptin (ng/ml)	-0.192 ± 0.650	-0.064	0.770	-1.322 ± 2.265	-0.116	0.565
Active ghrelin (pg/ml)	-1.260 ± 1.096	-0.227	0.262	2.511 ± 3.913	0.120	0.527
Validation study (N=33)						
Fasting insulin (pg/ml)	-0.025 ± 0.021	-0.216	0.244	-0.026 ± 0.125	-0.037	0.840
C-peptide (ng/ml)	-13.446 ± 9.833	-0.243	0.182	-12.840 ± 55.623	-0.042	0.819
Fasting glucose (mmol/L)	3.795 ± 2.642	0.258	0.162	18.956 ± 14.586	0.232	0.204
IGF-1 (ng/ml)	-0.428 ± 0.959	-0.103	0.661	3.568 ± 5.487	0.140	0.523
Leptin (ng/ml)	-0.265 ± 0.228	-0.222	0.254	-2.182 ± 1.215	-0.328	0.083
Active ghrelin (pg/ml)	-0.138 ± 0.612	-0.040	0.823	-4.984 ± 4.804	-0.191	0.308

Data are adjusted for sex and age.
